# Breast Reconstruction With Subpectoral, Air-Filled Tissue Expander Following Nipple-Sparing Mastectomy: A Safe Alternative to Saline-Filled Tissue Expanders

**DOI:** 10.7759/cureus.81520

**Published:** 2025-03-31

**Authors:** Ryan A Cantrell, Alexander L Mostovych, Claire Fell, Quinton L Carr, Colton H Connor, Sierra M Shockley, Lucas J Niebrugge, Shriya D Dodwani, Alexander T Nixon, Bradon J Wilhelmi

**Affiliations:** 1 Plastic and Reconstructive Surgery, University of Louisville School of Medicine, Louisville, USA; 2 Plastic and Reconstructive Surgery, Vanderbilt University Medical Center, Nashville, USA

**Keywords:** air-filled, breast reconstruction, nipple-sparing mastectomy, subpectoral, tissue expanders

## Abstract

Background: Compared with immediate implant-based reconstruction, staged reconstruction using tissue expanders has been reported to have decreased nipple and mastectomy flap necrosis. Immediate filling of the expander with saline can place unnecessary pressure on the mastectomy flaps, increasing the risk of ischemia. Tissue expanders come packaged pre-expanded with air. We propose using tissue expanders with factory air at the index surgery to optimize nipple positioning and decrease skin and nipple necrosis; this also allows the draping of redundant skin to prevent skin wrinkling and nipple retraction.

Methods: A single-center, retrospective, and single-surgeon cohort study of 53 patients (91 breasts) was performed. Patients included in the study underwent nipple-sparing mastectomy (NSM) followed by immediate two-staged implant-based breast reconstruction. Patient demographics and tissue expander-associated complications were abstracted from electronic medical records.

Results: Of the 53 patients, the most frequent complication was wound dehiscence, occurring in three patients (5.7%). Less common, self-limited complications included one case of hematoma and one case of seroma (1.9% each). There was also one reported tissue expander infection (1.9%). There were no incidents of other postoperative complications of interest, such as skin flap necrosis, nipple necrosis, nipple-areola complex (NAC) malposition, tissue expander malposition, and adjuvant treatment delay.

Conclusion: Our results indicate subpectoral placement of tissue expanders, with the manufacturer's original air still intact, did not result in NAC or mastectomy flap necrosis in any patient. Additionally, there were no instances of NAC or tissue expander malpositioning, nor were there delays in preplanned adjuvant cancer treatment. Furthermore, the absence of complications specifically associated with air-based tissue expander placement underscores the safety of this technique and supports its continued clinical use.

## Introduction

According to the American Society of Plastic Surgeons, 6.8 million reconstructive procedures were performed in 2020, including 137,808 breast reconstructive procedures (a 1% increase from 2019, and a 75% increase since 2000) [1]. Of the reported breast reconstructions, 83,487 procedures (60%) consisted of a tissue expander and implant reconstruction, and with regard to the timing of reconstruction, 105,665 procedures consisted of direct-to-implant reconstruction (76.7%) [1]. It has been demonstrated that patients who undergo breast reconstruction reported significantly higher scores for cosmetic outcome, overall body satisfaction, and breast satisfaction, compared with patients who had forgone reconstruction [2,3].

Breast reconstruction can be further classified based on the anatomical plane of implant placement, either subpectoral (dual-plane) or prepectoral, with studies demonstrating comparable complication rates between the two approaches [4]. An advantage of tissue expander-to-implant breast reconstruction is that the final implant size can be made larger in volume than a patient’s native breast size, and this has been shown to correlate with patient satisfaction without an increase in complications [5]. Two-stage breast reconstruction provides an opportunity to address rotation or displacement of the reconstructed breast at definitive implant exchange [6].

We propose avoiding the filling of the tissue expander with saline during the initial intraoperative period, instead maintaining the tissue expander with its factory air. This air-filled tissue expander approach is lighter and places less pressure on the mastectomy skin flaps, decreasing the risk of flap and nipple necrosis. Using air within the tissue expander maintains an even distribution throughout the expander, avoids inferior pooling and added weight on inferior mastectomy flaps, maintains expander positioning, and improves nipple positioning. The patient is left with a breast mound at the time of mastectomy. Skin is smoothly draped over the air-filled expander, providing more acceptable initial results and easier secondary pocket development.

## Materials and methods

Design and study population

A single-center, retrospective, and single-surgeon cohort study was performed. Patient inclusion criteria consisted of undergoing nipple-sparing mastectomy (NSM) followed by immediate two-stage subpectoral implant-based breast reconstruction. The procedures included in the study occurred between January 2020 and July 2023. Exclusion criteria involved patients who underwent a skin-sparing mastectomy, radical mastectomy, direct-to-implant reconstruction, pre-pectoral tissue expander placement, or those lost to follow-up. During the period 2020-2023, the senior surgeon exclusively used air-filled tissue expanders. Thus, all patients fitted with these tissue expanders who met the inclusion criteria were included in this study.

**Figure 1:**
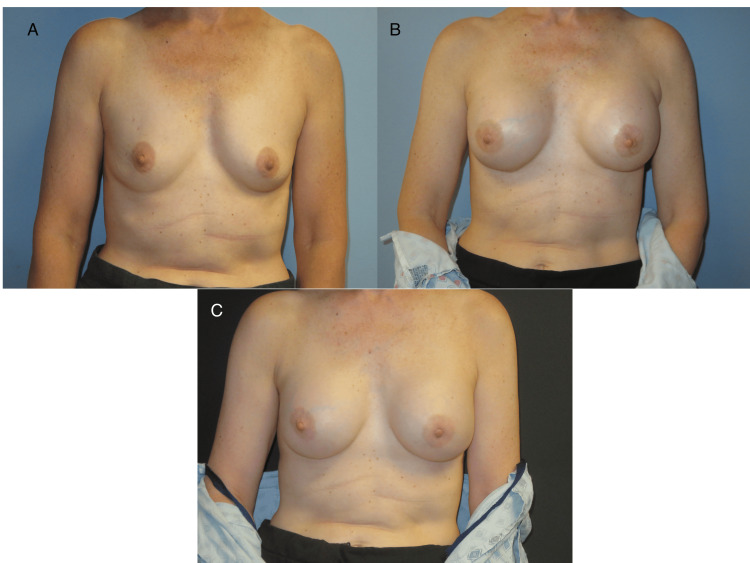
First patient's images The first patient’s post-operative images are presented in sequence, beginning with Image A, which depicts the preoperative period before NSM, followed by the placement of subpectoral, air-filled tissue expanders. Image B shows the patient with subpectoral, air-filled tissue expanders in place, awaiting the expander-to-implant stage of reconstruction. Image C shows the patient postoperatively, following the expander-to-implant stage of reconstruction. NSM: nipple-sparing mastectomy

**Figure 2:**
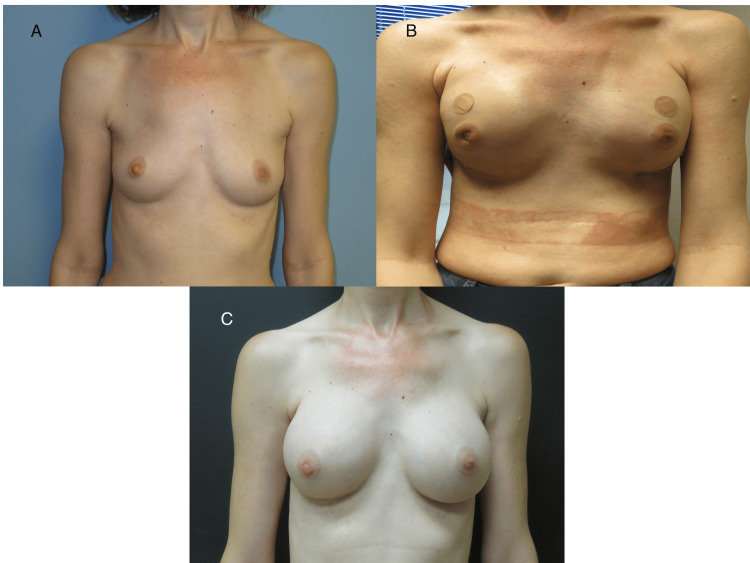
Second patient's images The second patient’s post-operative images are presented in sequence, beginning with Image A, which depicts the preoperative period before NSM, followed by the placement of subpectoral, air-filled tissue expanders. Image B shows the patient with subpectoral, air-filled tissue expanders in place, awaiting the expander-to-implant stage of reconstruction. Image C shows the patient postoperatively, following the expander-to-implant stage of reconstruction. NSM: nipple-sparing mastectomy

**Figure 3:**
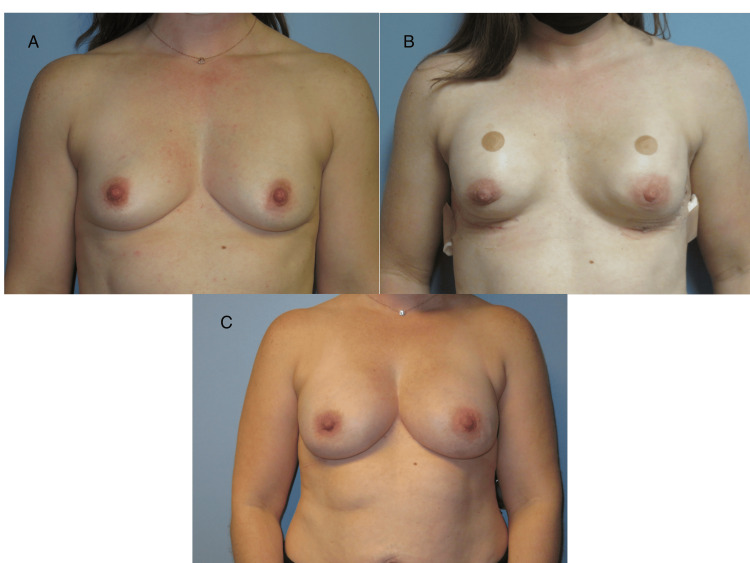
Third patient's images The third patient’s post-operative images are presented in sequence, beginning with Image A, which depicts the preoperative period before NSM, followed by the placement of subpectoral, air-filled tissue expanders. Image B shows the patient with subpectoral, air-filled tissue expanders in place, awaiting the expander-to-implant stage of reconstruction. Image C shows the patient postoperatively, following the expander-to-implant stage of reconstruction. NSM: nipple-sparing mastectomy

Patient images, demographics, and clinical outcomes were collected through the review of electronic medical records. Preoperative images, images following NSM with immediate tissue expander placement, and images taken during the final expander-to-implant stage of reconstruction were collected from three different patients and assembled into Figures 1, 2, 3. All the patients with images included in this study have been provided informed consent on the use of their images and granted the use of their images for scientific publications. Patient demographic variables obtained and assembled into the table include age, BMI, obesity status, and comorbidities (diabetes, smoking status, and hypertension). Patient clinical outcomes of interest include tissue expander infection, wound dehiscence, hematoma, seroma, skin flap necrosis, nipple necrosis, nipple-areola complex (NAC) malpositioning, tissue expander malpositioning, and adjuvant treatment delay; these results were assembled into the table. Data management and presentation were conducted using Microsoft Excel. Patient demographics and postoperative complications were organized into tables and reported as percentages of the study population. To contextualize our complication rates, these averages were compared with those reported in the existing literature.

Operative technique

The temporary skin closure left by the oncologic breast surgeon following a NSM performed via an inframammary approach is reopened by removing the staples. A subpectoral pocket is created using electrocautery to raise the pectoralis major along the inframammary fold. Once the pectoralis major is released inferiorly, it is elevated primarily through blunt and digital dissection, while the pectoralis minor remains in its anatomic position. The medial attachments of the pectoralis major are largely left intact.

A textured, three-tab tissue expander with an integrated port is preferred (Mentor Worldwide LLC, Johnson & Johnson). The implant is bathed in a triple-antibiotic solution containing vancomycin, cefazolin, and gentamicin prior to insertion. The tissue expander is inserted with the factory-placed air remaining within the tissue expander. Cannulation of the integrated port is avoided to prevent air loss from the tissue expander during the perioperative period. The tissue expander is secured in the subpectoral pocket using the inferior and lateral tabs with 2-0 PDS sutures, ensuring adequate positioning by securing it to healthy tissue and the rib periosteum. The pectoralis major is draped over the tissue expander and advanced caudally, creating a dual-plane subpectoral pocket with the upper two-thirds of the expander covered by the pectoralis major muscle. Running 2-0 PDS suture is used to secure the free margin of the pectoralis major over the expander infero-laterally, preventing superior retraction of the muscle with what we call "anti-window shade" sutures; the tissue expander remains in a partial submuscular pocket at its superolateral margins. The serratus anterior muscle and its overlying fascia are left undisturbed within the submuscular pocket. One 19-French Blake drain is placed in each breast pocket, exiting the skin at the level of the mid-axillary line and the inframammary fold. The drains are draped superiorly between the pectoralis major and superior mastectomy flap, terminating inferomedially. Drains are secured to the skin using 2-0 nylon sutures. The superior mastectomy skin flap is re-draped in its anatomic position over the pectoralis major and tissue expander. A magnet is used to ensure the integrated tissue expander port is appropriately positioned, superior to the NAC. The skin is closed in layers using interrupted 2-0 PDS deep dermal sutures, followed by 3-0 PDS running subcuticular sutures. At two weeks postoperatively, the air within the tissue expander is exchanged for saline to allow for vascular recovery of the mastectomy skin and NAC. Expansions are then performed weekly until the desired volume is reached, based on patient preference and soft tissue compliance.

## Results

A total of 53 patients met the inclusion criteria for our study. Of the 53 patients, 38 (71.7%) underwent bilateral NSM and the remaining 15 (28.3%) received a unilateral NSM, totaling 91 breasts. Demographic data revealed a mean patient age of 49.6 years, with 12 of 53 patients (22.6%) classified as obese, defined by a body mass index (BMI) greater than 30.0 (Table 1). When asked about smoking status, 13 (24.5%) reported being former smokers, while only four (7.5%) were current smokers. Identified comorbidities in the cohort included diabetes mellitus in six patients (11.3%) and hypertension in 18 patients (34.0%).

**Table 1:** Patient demographics BMI: body mass index

	Patients (n=53)
Mean age, year (standard deviation)	49.6 (9.6)
Mean BMI, kg/m² (standard deviation)	26.36 (4.3)
Obesity, no. (%)	12 (22.6)
Tobacco use, no. (%)	
- Former use	13 (24.5)
- Current use	4 (7.5)
Comorbidities, no. (%)	
- Diabetes mellitus	6 (11.3)
- Hypertension	18 (34.0)

Postoperative complications occurring during the period immediately following NSM and tissue expander implantation, up until the expander-to-implant second stage of reconstruction, were reported (Table 2). The most frequent complication among patients was superficial incisional wound dehiscence without implant exposure, occurring in three patients (5.7%), all of whom were managed conservatively with frequent local wound care and close outpatient follow-up. All three patients had undergone NSM via a lateral radial incision, a technique that is now less commonly utilized at our institution. Less common, self-limited complications included one case of hematoma and one case of seroma (1.9% each). One case of tissue expander infection was reported (1.9%), which necessitated explantation. There were no incidents of other postoperative complications of interest, such as skin flap necrosis, nipple necrosis, NAC malposition, tissue expander malposition, or delay in initiation of adjuvant treatment.

**Table 2:** Postoperative complications NAC: nipple areola complex

Tissue expander infection, no. (%)	1 (1.9)
Wound dehiscence, no. (%)	3 (5.7)
Hematoma, no. (%)	1 (1.9)
Seroma, no. (%)	1 (1.9)
Skin flap necrosis, no. (%)	0 (0)
Nipple necrosis, no. (%)	0 (0)
NAC malposition, no. (%)	0 (0)
Tissue expander malposition, no. (%)	0 (0)
Adjuvant treatment delay, no. (%)	0 (0)

## Discussion

According to the American Society of Plastic Surgeons, 60% of reported breast reconstructions implement tissue expanders [1]. Thus, with such a high patient population evidence-based optimization of two-staged breast reconstruction procedures should be highly regarded to improve women’s quality of life and overall outcomes.

All surgical procedures including two-staged breast reconstruction carry a risk of complications. In the case of expander-to-implant breast reconstruction, patients may experience mastectomy flap necrosis, NAC necrosis, delays to adjuvant therapy, and decreased quality of life, all of which are targeted for decreased incidence with the method proposed in this manuscript. As high as 15.3% of breasts experience mastectomy flap necrosis during breast reconstruction [7]. Postmastectomy flaps are highly vulnerable to decreased perfusion, and multiple risk factors, such as intraoperative fill volume (namely tumescence), smoking status, age, hypertension, and obesity, have been linked to flap necrosis [8]. Mastectomy flap and NAC necrosis can lead to considerable challenges for the patient, including wound management problems, suboptimal aesthetic outcomes, implant extrusion, patient distress, financial loss, and, among the most feared complications, delays in adjuvant treatment [9,10]. It is also worth mentioning the association of chronic wounds, such as those found with surgical complications, with patients reported decreased quality of life [11-13]. Thus, with the large patient volume and devastating associated complications, any actionable techniques to improve outcomes in tissue expander breast reconstruction are of utmost importance.

Traditionally, during the intraoperative period, surgeons remove the factory air and inject saline into the tissue expander. More recently, some surgeons have even introduced a set volume of air (dependent on the institution) into the tissue expander [14,15]. We propose leaving the manufacturer air in the tissue expander during the intraoperative period to prevent mastectomy flap necrosis, NAC necrosis, and incision site complications. Air is less dense than saline, therefore, it is theorized that air-filled tissue expanders will mitigate excessive outward stress on the mastectomy flap and incision site. Excessive force imparted by a traditionally saline-filled tissue expander can interrupt the subdermal plexus supplying the mastectomy flap and NAC resulting in necrosis [8,16,17]. In addition, larger tissue expander volumes, which impart a greater outward force, can be linked to increased complication risks and vascular compromise [16,18,19].

Air-filled tissue expanders have yet another advantage over saline-filled ones in that air fills uniformly and does not collect at the bottom of the expander like saline [14]. Uniform air inflation in the tissue expanders will impart uniform outward force preventing uneven pressure gradients on mastectomy flaps, NAC, and/or incision sites [14]. In addition, uniform air inflation and force are postulated to be associated with a more satisfactory NAC placement, mitigating unwanted nipple positioning. Optimal nipple placement in breast reconstruction has already been linked to the use of tissue expanders, thus, air-filled tissue expanders have the potential to further optimize NAC placement [20]. Furthermore, prior studies have reported that the critical time period when patients most often experience mastectomy flap necrosis is within the first two weeks following tissue expander placement [21]. This finding is another added benefit of our method of intraoperative air tissue expanders. At our institution, no patients will begin serial saline dilations in the clinic until they are out of this two-week window, allowing for adequate revascularization of the tissue before subsequent dilation. This serial transition from air to saline in the postoperative period allows for tissue adjustment to weighted tissue expanders, facilitating a more seamless tissue expander-to-implant exchange in the second stage of reconstruction.

Prior research studies have investigated the use of air in expander-to-implant breast reconstruction as a means of avoiding tissue necrosis and other complications [14,22]. However, there are several key differences between the techniques in the previously published studies and the one described in our study. Becker et al. reported using air in prepectoral tissue expanders in conjunction with acellular dermal matrix (ADM) [14]. One of the largest differences between this study and many of the others is that our institution uses subpectoral tissue expander placement rather than prepectoral. The reported added benefit of prepectoral implantation is less patient-reported pain immediately following surgery, while there is no difference in long-term patient-reported outcomes [22]. In addition, Nelson et al. found prepectoral placement was associated with a higher rate of seromas [22]. In our study, tissue expanders were placed under the pectoralis major in an effort to push the expander against the chest wall reducing pressure on the NAC and maintaining a centered location of the tissue expander under the NAC. Subpectoral tissue expander placement is the technique of choice due to our institution's patient population on average having one or more of the following significant comorbidities: radiation, smoking, or poorly perfused mastectomy flaps [23]. Additionally, in a study by Becker et al., the tissue expanders were only filled from 0%-50% with air. In contrast, in our study, the manufacturer’s air left in the tissue expander was not modified intraoperatively, nor was the expander cannulated. Another difference in our study is that many of the currently published studies never transition the air-filled tissue expanders to saline, while in this study, the air is serially exchanged for saline in subsequent clinic follow-ups prior to tissue expander exchange for breast implants [14-16]. Some of the results reported in the currently published studies on air-filled tissue expanders have mixed conclusions on their efficacy. Some studies, such as Yesantharao et al., have found a protective effect of using air [16]. In contrast, other studies reported no significant advantage of using air-filled expanders [24,25].

To contextualize our findings and assess their relevance, a PubMed search was conducted. The two complication variables of most interest include NAC and skin flap necrosis. Thus, we searched PubMed from 2019-2024, including keywords "NSM," "NAC necrosis," and "skin flap necrosis" in the titles or abstracts. A study was included only if NAC necrosis and/or skin flap necrosis was reported in association with patients undergoing NSM followed by implant-based breast reconstruction. Studies outside the 2019-2024 search window were included if they were found in the references section from the initial literature review. These results were assembled in Table 3.

**Table 3:** Literature complications NAC: nipple-areola complex

Manuscript title	Authors	Year of publication	Mastectomy flap necrosis (%)	NAC necrosis (%)
Nipple-areolar complex ischemia and necrosis in nipple-sparing mastectomy	Ahn et al. [26]	2018	-	31.3
Ischemic complications after bilateral nipple-sparing mastectomy and implant-based reconstruction: a critical analysis	Razavi et al. [7]	2021	15.3	30.6
Prepectoral versus subpectoral breast reconstruction after nipple-sparing mastectomy: a systematic review and meta-analysis	Nolan et al. [27]	2024	6.5	4.7
Direct-to-implant versus immediate free flap reconstruction after nipple-sparing mastectomy: a propensity score-matched analysis	Abdou et al. [28]	2023	8.3	6.5

Our investigation found that the patients receiving NSM with immediate air-filled tissue expander reconstruction experienced no complications to date related to skin necrosis, NAC necrosis, tissue expander malposition, or NAC malposition (Table 2). Prior studies that reported on the complication rates following NSM with tissue expander reconstruction report higher rates of NAC necrosis [26,27]. The rate of NAC necrosis reported in the literature is variable with some publications reporting necrosis rates as high as 31.3% and others as low as 4.7% [26,27]. The literature also reports skin flap necrosis rates as high as 15.3% [7]. It is important to note that these complication rates were from studies in which patients underwent NSM with immediate saline-filled tissue expander reconstruction. These necrosis complication rates prompted the senior author to cease intraoperatively filling tissue expanders with saline to reduce pressure on the NAC and skin flaps, thereby improving wound healing. Additionally, the tissue expanders are placed in a subpectoral fashion to further decrease pressure on the NAC and skin flaps. The study identified no instances of malpositioning of NACs or tissue expanders, which we attribute to the reduced air-filled weight of the expanders, possibly preventing the loosening of tabs or sutures. We also believe that heavy saline-filled tissue expanders, when placed immediately, do not allow sufficient time for healing and possibly increase the risk of malpositioning. Malposition is not reported frequently in the literature, but Chen et al. reported asymmetry in 4.3% of patients undergoing NSM with immediate saline-filled tissue expander reconstruction [29]. An important implication of air-filled tissue expanders being associated with decreased complication rates is ensuring timely initiation of adjuvant cancer treatment for patients, preventing delays due to reconstructive complications. All the patients in our study who were indicated to receive adjuvant cancer therapy began their treatment on time. Often, reconstructive surgical complications may delay adjuvant treatment; this finding is reported by Huttunen et al., who concluded that patients undergoing immediate breast reconstruction were more likely to receive adjuvant treatment later than those undergoing mastectomy alone [30].

This study is not without its limitations. This is a single-institution retrospective cohort study limiting external validation. The retrospective nature may introduce biases and inaccuracies in data reporting and collection. The study is also likely underpowered, having identified 53 patients or 91 breasts, which may limit the statistical significance of the findings. Future research would benefit from larger, prospective, multicenter studies to more definitively assess the utilization and safety of air-filled tissue expanders. Nonetheless, air-filled tissue expanders have been a staple at this institution for several years. The senior author’s expertise may provide valuable insights into this technique.

## Conclusions

Following NSM, two-stage alloplastic breast reconstruction using tissue expanders is a well-established technique. Our results indicate that subpectoral placement of tissue expanders, with the manufacturer's original air still intact, did not result in NAC or mastectomy flap necrosis in any patient. Additionally, there were no instances of NAC or tissue expander malpositioning, nor were there delays in preplanned adjuvant cancer treatment. Furthermore, the absence of complications specifically associated with air-based tissue expander placement underscores the safety of this technique and supports its continued clinical use.
